# The absolute number of small and diminutive adenomas with high-grade dysplasia is substantially higher compared with large adenomas: a retrospective pooled study

**DOI:** 10.3389/fonc.2024.1294745

**Published:** 2024-02-12

**Authors:** Jiancheng Zhang, Huajun Sun, Fei Xiong, Shan Lei, Guanyu Zhou, Xun Xiao, Lin Liu, Pu Wang

**Affiliations:** ^1^ Department of Gastroenterology, Sichuan Provincial People’s Hospital, University of Electronic Science and Technology of China, Chengdu, China; ^2^ Department of Pathology, Sichuan Provincial People’s Hospital, University of Electronic Science and Technology of China, Chengdu, China; ^3^ Institute of Natural Sciences, MOE-LSC, School of Mathematical Sciences, CMA-Shanghai, and SJTU-Yale Joint Center for Biostatistics and Data Science, Shanghai Jiao Tong University, Shanghai, China and Shanghai Artificial Intelligence Laboratory, Shanghai, China

**Keywords:** colorectal cancer, CRC screening, small polyp, high-grade dysplasia (HGD), composition ratio

## Abstract

**Introduction:**

The risk that a large polyp (≥10 mm) evolves into high-grade dysplasia (HGD) is relatively high compared with that of a small/diminutive polyp (<10 mm). Recently, the detection of small and diminutive polyps has been substantially improved with the advancement of endoscopy. However, further research is needed on the role of the incidence of HGD caused by the co-occurrence of small and diminutive polyps in the progression of HGD. In this study, we aim to investigate whether and how the small and diminutive polyps correlate with the incidence of HGD in the population.

**Methods:**

The pooled data were deeply analyzed from four published randomized controlled trials (RCTs) regarding colon polyp detection. All polyps detected were examined and confirmed by pathologists. The primary outcome was the composition ratio of the HGD polyps in each polyp size category.

**Results:**

Among a total of 3,179 patients with 2,730 polyps identified, there were 83 HGD polyps confirmed, and 68 patients had at least one polyp with HGD. The risk of development of HGD was lower for a single small and diminutive polyp than for one large polyp (2.18% vs. 22.22%, *P* < 0.0001). On the contrary, the composition ratio for HGD from small and diminutive polyps was significantly higher than that from the large ones (68.67% vs. 31.33%, *P* < 0.0001). The combined number of HGD presented a trend negatively correlated to size.

**Conclusions:**

Our data demonstrated that the absolute number of HGD significantly derives more from small and diminutive polyps than from the large ones, and the collective number of small and diminutive polyps per patient is indicative of his/her HGD exposure. These findings positively provide novel perspectives on the management of polyps and may further optimize the prevention of colorectal cancer.

**Systematic Review Registration:**

http://www.chictr.org.cn, identifier ChiCTR1900025235, ChiCTR1800017675, ChiCTR1800018058, and ChiCTR1900023086.

## Introduction

Considerable evidence demonstrates that there is a close correlation between polyps and colorectal cancer (CRC) (1–12). High-grade dysplasia (HGD), characterized by marked complex glandular crowding and irregularity of glands, cribriform architecture, and intraluminal necrosis as architectural features, is one of the characteristics of advanced adenomas and is associated with an increased risk of developing CRC (13). In addition, more than 90% of colon cancers are adenocarcinomas, with HGD being the main precursors (13–17). Thus, it is critically important to identify patients who are more prone to CRC among the endoscopic screening cohort (10, 18). It has been well-acknowledged that there is a strong correlation between the diameter of colorectal polyps and the development of CRC (19–23). For instance, a study has shown that large-size polyps account for 51.1% of cases diagnosed as malignancy or HGD via histopathology, versus 18.7% from small and diminutive polyps (24). Similarly, Vleugels J.L.A. et al. concluded that polyps <10 mm in diameter have a lower fraction of malignancy transformation than large ones (25), even if it is considered to be neglectable for sigmoid and rectum colon (15). More recently, the detection rate of colon polyps has increased as colonoscopy advances the sensitivity and accuracy of detecting small and diminutive polyps (26), especially with artificial intelligence (AI), i.e., computer-aided detection (CADe), in identifying small and diminutive polyps in a real-time manner (27, 28). Following extensive literature research, it is noted that there are substantially large numbers of small and diminutive polyps detected, yet the contribution of small and diminutive polyps to the incidence of HGD is scarcely reported (19, 24, 29).

In the current study, we retrospectively investigated the constitution of HGD cases according to different size categories of polyps, aiming to provide more perspective on the management of polyps of different sizes and HGD.

## Materials and methods

### Pooled analysis

We collected data from four prospective randomized controlled trials (RCTs) conducted at the Endoscopy Center of Sichuan Provincial People’s Hospital, China, from September 2017 to September 2019. Three of the studies were two-arm trials aiming to analyze the adenoma detection rate (ADR) (27, 30, 31), while the fourth was a two-arm tandem trial focusing on the adenoma miss rate (AMR) (32). The trials employed the same CADe System (EndoScreener, Wision A.I., China) as the AI intervention. In the four trials, all detected polyps were taken cold forceps biopsy or resected by snare polypectomy and pathologically diagnosed according to WHO standards (13). All colonoscopy procedures were performed by experienced endoscopists, and the diameter of the polyps was measured based on the endoscopists’ judgment by comparing fully opened biopsy forceps during colonoscopy procedures. All pathological diagnoses in four studies were performed by two pathologists, first diagnosed by one registrar and then reviewed by one staff specialist. Pathological slides containing HGD were then selected and confirmatory checked by the third pathologist according to WHO standards. Precancerous polyps and non-neoplastic polyps detected in these four RCTs were included, such as sessile serrated lesions (SSLs) and traditional serrated adenomas (TSAs), and polyps with invasive cancer were all excluded. The HGD diagnostic criteria for conventional adenomas were based on the definition of the WHO (2019, 5Th). In SSL, cases with serrated dysplasia, high-grade intestinal dysplasia, were regarded as SSLD and were included in the HGD group in the study, due to the high and rapid progression to carcinoma (33).

In this study, diminutive polyps refer to polyps with a diameter ≤5 mm, small polyps refer to those with a diameter of 6–9 mm, and large polyps refer to those ≥10 mm. The primary outcome was the composition ratio of the HGD polyps in each polyp size category, defined as the number of polyps containing HGD within each size category divided by the total number of polyps containing HGD. Notably, if a patient is found to have both large and small/diminutive polyps with HGD, this patient will be categorized under the group of patients with large-sized polyps with HGD at the patient-level analysis. Our secondary outcome is the absolute proportion of HGD between small/diminutive and large polyps, which was determined by dividing the number of polyps with HGD by the total number of polyps in each respective size category.

### Statistical analysis

The two-sample proportion test was used as the primary endpoint is the compositional ratio between the HGDs found in small/diminutive polyps and large polyps, and it fitted the assumptions of the two-sample proportion test. The null hypothesis of the two-sample proportion test was that two proportions were equal, and we could reject the null hypothesis and say that the two compared proportions were significantly different when the *P*-value was less than 0.05.

Statistical analysis was performed with R studio V.3.4.0. Regarding the composition ratio of HGD in screening and diagnostic populations, the two-sample proportion test was conducted for statistical difference. Comparison of the HGD ratios between different size categories was performed using the *χ* ^2^ test for categorical variables. A two-sided *P*-value of 0.05 was used as the threshold for statistical significance. Linear analysis was applied in this study.

### Ethical approval statement

All procedures performed in this study involving human participants were under the ethical standards of the institutional IRB and with the Helsinki declaration.

### Patient consent statement

Due to the retrospective nature of the study, the Institutional Review Board (IRB) of the Sichuan Academy of Medical Sciences & Sichuan Provincial People’s Hospital waived the need to obtain informed consent.

The protocols of the stated four trials were approved by the IRB of the Sichuan Academy of Medical Sciences & Sichuan Provincial People’s Hospital. The trials were registered with the Chinese Clinical Trial Registry (http://www.chictr.org.cn) (Registration Nos.: ChiCTR1900025235, ChiCTR1800017675, ChiCTR1800018058, and ChiCTR1900023086, respectively). Written informed consent was obtained from all participants before the colonoscopy procedure in the four trials.

## Results

The four well-designed RCTs were two-arm designed and performed with large prospective cohorts in China, describing the improvement of ADR after deploying a novel CADe system during colonoscopy.

### Baseline data

There were 3,179 patients included from four trials, and 2,730 polyps were detected, biopsied, and pathologically diagnosed, among which 83 (83/2,730, 3.04%) were found to have pathological evidence of HGD and 68 patients were found to have at least one polyp with HGD (**Table 1**). The classification of the polyp and the patient baseline details are shown in **Supplementary Figures S1**, **S2** and **Supplementary Table S1**.

**Table 1:** Baseline data.

	Study 1^a^	Study 2^b^	Study 3^c^	Study 4^d^	Total
Number of centers	1	1	1	1	4
Study design	2-arm RCT	2-arm tandem^e^ RCT	2-arm RCT	2-arm RCT	/
Including criteria	Screening/diagnostic/surveillance colonoscopy	Screening/diagnostic/surveillance colonoscopy	Screening/diagnostic/surveillance colonoscopy	Screening/diagnostic/surveillance colonoscopy	/
Population	Asian	Asian	Asian	Asian	Asian
Age range	≥18	18–75	18–75	18–75	/
Patients enrolled	1,058	369	790	962	3,179
Polyp detected	767	529	625	809	2,730
HGD adenoma^f^	31	3	21	28	83
HGD patients^g^	27	3	17	21	68
Published date	2019	2020	2020	2020	/

a Wang P, Berzin TM, Glissen Brown JR, et al. Real-time automatic detection system increases colonoscopic polyp and adenoma detection rates: a prospective randomised controlled study. Gut. 2019; 68:1813-1819.

b Wang P, Liu P, Glissen Brown JR, et al. Lower Adenoma Miss Rate of Computer-Aided Detection-Assisted Colonoscopy vs Routine White-Light Colonoscopy in a Prospective Tandem Study. Gastroenterology. 2020;159(4):1252-1261.e5.

c Liu P, Wang P, Glissen Brown JR, et al. The single-monitor trial: an embedded CADe system increased adenoma detection during colonoscopy: a prospective randomized study. Therap Adv Gastroenterol. 2020; 13:1756284820979165.

d Wang P, Liu X, Berzin TM, et al. Effect of a deep-learning computer-aided detection system on adenoma detection during colonoscopy (CADe-DB trial): a double-blind randomised study. Lancet Gastroenterol Hepatol. 2020;5(4):343-351.

e Two the same day back-to-back colonoscopy procedures.

f Number of adenomas with HGD.

g Number of patients with at least one HGD adenoma.

### The prevalence of HGD at the polyp level

**Figure 1:**
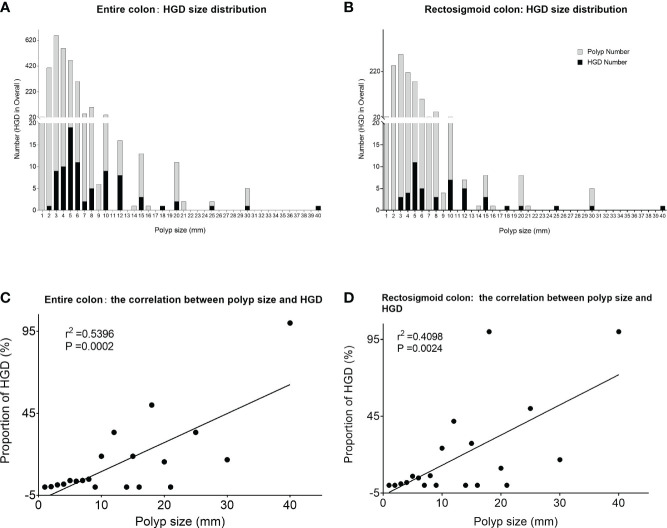
Size distribution and proportion of HGD polyps at the per-polyp level. **(A)** The distribution of HGD among all confirmed polyps of different sizes in the entire colon. **(B)** The distribution of HGD among all confirmed polyps of different sizes within the rectosigmoid colon. **(C)** Significant positive correlation between polyp size and HGD in the entire colon. **(D)** The significant positive correlation between polyp size and HGD within the rectosigmoid colon. *HGD, high-grade dysplasia.

From a single polyp point of view, large polyps are more likely to harbor HGD compared with small and diminutive polyps. The risk of HGD for a single polyp is positively correlated with polyp size (*r* ^2 ^= 0.5396, *P* = 0.0002), and the positive correlation also holds on the sigmoid colon and rectum (*r* ^2 ^= 0.4098, *P* = 0.0024) (**Figure 1**).

Moreover, polyps with villous or tubulovillous features have a higher risk of HGD, 33.33% and 44.44%, respectively, compared with the other histology features (**Table 2A**). Within villous-structured polyps, larger ones are more likely to contain HGD in total. These data are consistent with the traditional view of assessing risk from the size of each polyp. On the contrary, from the perspective of composition ratio, which means the absolute number of HGD contributors, far more HGDs came from non-villous polyps (**Table 2B**). Due to the significantly higher quantity of small and diminutive polyps compared with large polyps, the total number of HGD contained within the small polyps far exceeds those within the large polyps. Similarly, the composition ratio of HGD derived from small and diminutive polyps was significantly higher compared with that from larger polyps (68.67% vs. 31.33%, *p* < 0.0001). Considering only rectosigmoid, there is also a trend that small/diminutive polyp groups contribute more HGD than large polyp groups (56.52% vs. 43.48%, *p* = 0.297) (**Table 3**). The composition ratio and individual risk of small/diminutive HGD polyps are detailed in **Supplementary Table S2**.

**Table 2A:** Histology characteristics of polyps.

Characteristics	Entire colon	Rectosigmoid colon
General		
Total number of polyps	2,730	1,225
Total number of adenomas	1,528	560
Total number of advanced adenomas	83	46
Detailed histology		
Villous	3	2
HGD	1 (33.33%)	1 (50.00%)
Tubulovillous	27	15
HGD	12 (44.44%)	8 (53.33%)
Tubular	1,408	497
HGD	65 (4.62%)	34 (6.84%)
SSL	78	39
HGD	3 (3.85%)	1 (2.56%)
Not specified^a^	12	7
HGD	2 (16.67%)	2 (28.57%)
Hyperplastic and inflammatory	1,202	665
HGD	0 (0.00%)	0 (0.00%)
Total	2,730	1,225

SSL, sessile serrated lesion, included in adenomas; HGD, high-grade dysplasia.

a Not specified: no specific morphological classification of adenomas, which refers to small adenomas with serrated morphology being difficult to be further classified as TSA or SSL, due to factors such as embedding direction and insufficient tissue. Therefore, the term “serrated lesions, unclassified” was used in the original pathological report.

**Table 2B:** The incidence and composition ratio of HGD in villous and non-villous polyps.

	Tubulovillous/villous, %	Non-villous, %	*P*-value*
Entire colon			
HGD incidence			
Diminutive polyp (1–5 mm)	28.57 (2/7)	1.73 (37/2,136)	0.0001
Small polyp (6–9 mm)	40.00 (4/10)	3.04 (14/460)	<0.0001
Large polyp (≥10 mm)	53.85 (7/13)	18.27 (19/104)	0.0106
Total	43.33 (13/30)	2.59 (70/2,700)	<0.0001
HGD composition ratio			
Diminutive polyp (1–5 mm)	5.13 (2/39)	94.87 (37/39)	<0.0001
Small polyp (6–9 mm)	22.22 (4/18)	77.78 (14/18)	0.0027
Large polyp (≥10 mm)	26.92 (7/26)	73.08 (19/26)	0.0023
Total	15.66 (13/83)	84.34 (70/83)	<0.0001
Rectosigmoid colon			
HGD incidence			
Diminutive polyp (1–5 mm)	50.00 (2/4)	1.65 (16/968)	<0.0001
Small polyp (6–9 mm)	50.00 (2/4)	3.43 (6/175)	0.0012
Large polyp (≥10 mm)	55.56 (5/9)	23.08 (15/65)	0.0978
Total	52.94 (9/17)	3.06 (37/1,208)	<0.0001
HGD composition ratio			
Diminutive polyp (1–5 mm)	11.11 (2/18)	88.89 (16/18)	<0.0001
Small polyp (6–9 mm)	25.00 (2/8)	75.00 (6/8)	0.1336
Large polyp (≥10 mm)	25.00 (5/20)	75.00 (15/20)	0.0044
Total	19.57 (9/46)	80.43 (37/46)	<0.0001

*P-value from two-sample proportion tests.

**Table 3:** Composition ratio and individual risk of HGD polyps.

Characteristics	Entire colon			Rectosigmoid colon		
	Small and diminutive polyp (<10 mm), %	Large polyp (≥10 mm), %	*P*-value*	Small and diminutive polyp (<10 mm), %	Large polyp (≥10 mm), %	*P*-value*
HGD composition ratio	68.67 (57/83)	31.33 (26/83)	<0.0001	56.52 (26/46)	43.48 (20/46)	0.2971
Individual risk of HGD	2.18 (57/2,613)	22.22 (26/117)	<0.0001	2.26 (26/1,151)	27.03 (20/74)	<0.0001

*P-value from two-sample proportion tests.

**Figure 2:**
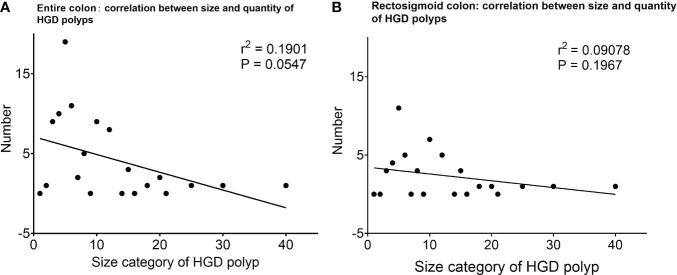
The number of HGD polyps at different size categories and their correlation. **(A)** The number of HGD polyps of different sizes, and the correlation analysis shows a trend that the number of HGD polyps is more related to small/diminutive polyps. **(B)** The number of HGD polyps of different sizes, and the correlation analysis shows a trend that the number of HGD polyps is more related to small/diminutive polyps within the rectosigmoid colon. *HGD, high-grade dysplasia.

Interestingly, from the collective point of view, a trend was seen in the number of HGD polyps that were negatively correlated with the polyp size category (*r* ^2 ^= 0.1901, *P* = 0.0547, **Figure 2**).

### The prevalence of HGD at the patient level

The composition ratio of patients with large HGD polyps is far lower than that of patients with only small and diminutive polyps (36.76% vs. 63.24%, *P* = 0.0036). Meanwhile, in the rectosigmoid colon, the composition ratio of patients with large HGD polyps did not show a significant difference from patients with only small and diminutive polyps (52.63% vs. 47.37%, *P* = 0.8185) (**Table 4**).

**Table 4:** Composition ratio and prevalence of patients with HGD polyp.

Characteristics	Entire colon			Rectosigmoid colon		
	Patients with small and diminutive polyp (<10 mm), %	Patients with large polyp (≥10 mm), %	*P*-value*	Patients with small and diminutive polyp (<10 mm), %	Patients with large polyp (≥10 mm), %	*P*-value*
Composition ratio	63.24 (43/68)	36.76 (25/68)	0.0036	47.37 (18/38)	52.63 (20/38)	0.8185
Prevalence	1.35 (43/3,179)	0.79 (25/3,179)	0.0382	0.57 (18/3,179)	0.63 (20/3,179)	0.8707

If a patient has both large and small polyps, he/she is classified into the large polyp group.

*P-value from two-sample proportion tests.

**Figure 3:**
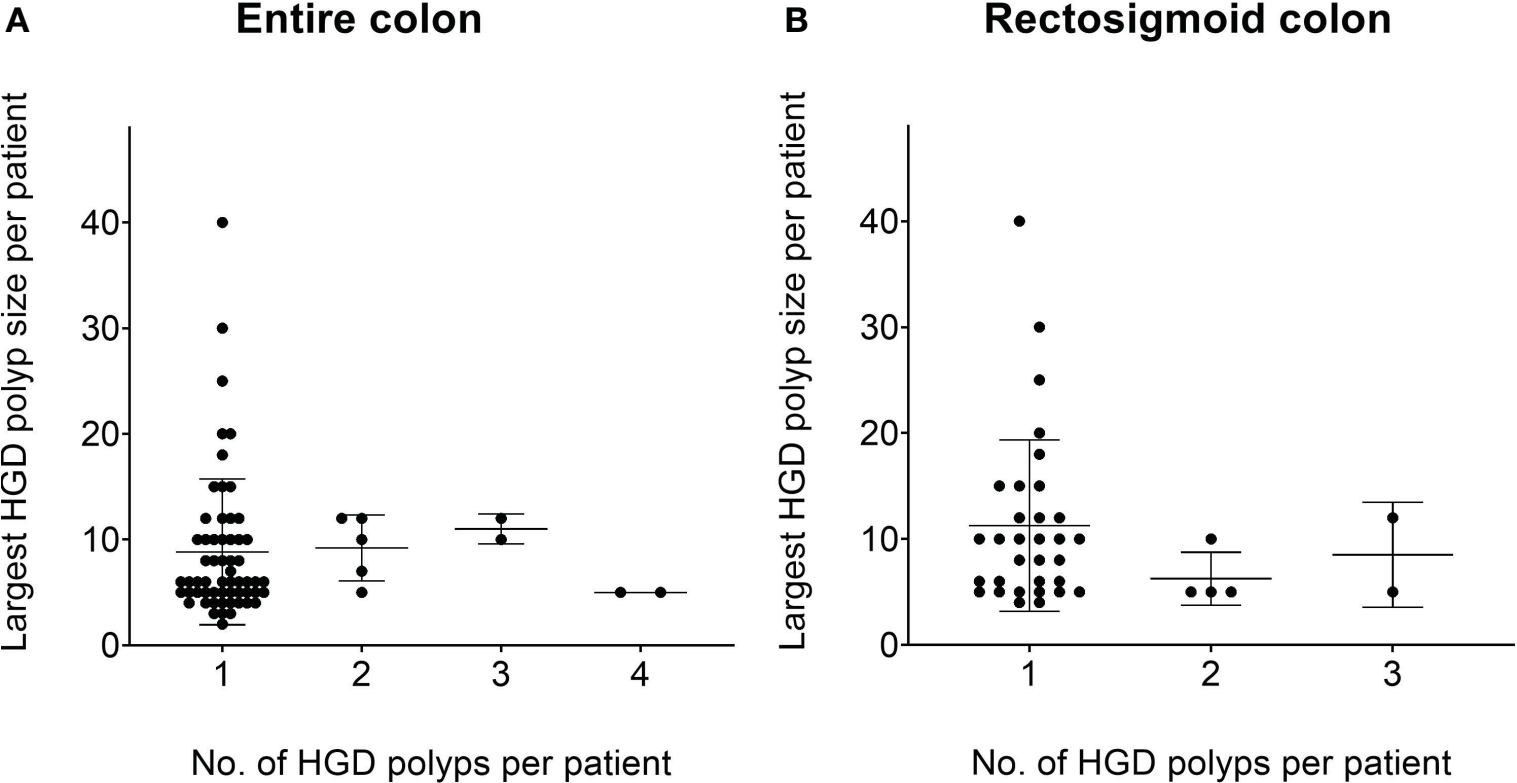
The number of HGD polyps at different size categories at the per-patient level. **(A)** The relationship between the average number of HGD polyps per patient and the size of HGD polyps per patient. It can be seen that the vast majority of patients with HGD polyps originate from a small/diminutive polyp. **(B)** A similar trend within the rectosigmoid colon. *HGD, high-grade dysplasia.

Our data show that the majority of patients with HGD polyp only have one small HGD polyp, which indicates that the abundance of small and diminutive polyps might be the stronger related factor for patients to develop HGD (**Figure 3**).

## Discussion

Our study revealed that, when considering the collective perspective, the majority of HGD incidences originated from small/diminutive and non-villous polyps. In addition, the combined number of HGD exhibited a trend of being negatively correlated with its size category. It is important to note that this does not conflict with the conventional viewpoint that large polyps are more likely to harbor HGD compared with small or diminutive ones (34). The conventional perception typically focuses on a single lesion, and our findings provide valuable insights into the cumulative risk associated with the abundance of small and diminutive polyps.

As well-documented in the literature among all adenomatous polyps (25), the ones with a diameter>=10mm, or containing villous histology, were generally considered equivalent indicators to the presence of HGD in determining a high-risk polyp (35). It is worth noting that the pathological nature of cancer is the high atypia of glands and cells (36), which is exactly what HGD represents. Therefore, we take HGD as the research object rather than subordinate features such as villous structure and polyp size. In addition, the concept of advanced adenoma is only a way to assess the risk of one single polyp and does not help to assess the overall risk of cancer in one patient, which leaves us to question the optimal approach to identifying high-risk patients. From the result of the study, a patient’s overall number of small and diminutive polyps emerges as a potential indicator of their risk for HGD presence and even the risk of CRC development, which is worthy of further investigation.

The small proportions of HGD within small and diminutive polyps have the potential to create a deceptive impression of safety, which could result in polyps being disregarded (35). Consequently, those polyps missed or untreated at the screening colonoscopy may be the cause of interval cancers, which account for approximately 8.2% of all CRC cases (1). In addition, patients whose polyps were removed during the screening colonoscopy but not pathologically evaluated have been advised a prolonged surveillance interval due to the unawareness of any possible HGD, which may also be the source of interval cancers due to the occurrence of metachronous advanced neoplasia during the prolonged surveillance interval (37–41). Our results might partially explain the interval CRC incidence in those days when small and diminutive polyps could not be effectively identified or treated.

The latest research further proves that with the application of high-definition imaging and CADe in colonoscopy, the number of small and diminutive polyps detected correctly has increased dramatically, and one issue was raised that the absolute number of HGD evolved from small and diminutive polyps is likely to be not lower than or even higher than large polyps both at the polyp and patient levels (16, 36, 42–44), which is very consistent with our results. Meanwhile, the neglect of small polyps is not only due to false sense induced by traditional statistical data focused on individual lesion risk but also due to the increasing application of optical biopsy (i.e., magnified narrow-band imaging) which allows endoscopists to predict pathological features via image enhanced endoscopy. Optical biopsy, recognized for its efficiency and cost-effectiveness, has gained increasing favor. In practice, endoscopists may often overlook small and diminutive polyps with a smooth surface, JNET 2A, III-L pit patterns, or lower classifications, as these clues usually indicate a low risk of malignant transformation (45–49). It is important to note that features on a plane such as mucosal microsurface structure and microvascular patterns have not been theoretically demonstrated to have the potential to achieve 100% one-to-one correspondence with stereoscopic histological features. Various studies have reported the accuracy of classification systems in the colon, such as Pit-Pattern, NICE, or JNET, ranging from 80% to 95%, with a notably low sensitivity of 40%–50% for advanced lesions (e.g., type 2B in the JNET system) (45–49). Consequently, optical diagnosis tends to underestimate the pathological characteristics of lesions (50–52), and any underestimation of pathological diagnosis may lead to the occurrence of interval colon cancer. In addition, there has been no universally recognized standard for the accuracy of predictions to be considered high enough in the long term. As a result of these limitations, these classification systems are currently employed as supporting tools to aid endoscopists in enhancing detection rather than serving as definitive diagnostic criteria to replace pathological diagnosis. Therefore, there is still controversy over whether to promote optical biopsy on a larger scale worldwide.

In current PIVI guidelines of ASGE, “diagnose-and-leave” and “resect-and-discard” strategies (53, 54) were recommended, from a population-based cost–benefit perspective, to treat optically diagnosed benign and diminutive polyps in the rectum and sigmoid colon, because these polyps are recognized to be less correlated with cancer development and metachronous cancer (34, 55, 56). However, the subgroup analysis in our study revealed a trend of a higher proportion of small-sized HGD not only in the entire colon but also in the sigmoid colon plus rectum. This finding suggests that an excessive disregard for small and diminutive polyps might pose a significant risk of interval cancer, even in the “safest colon segments”.

Furthermore, even from a cost-effectiveness and health economics perspective, the economic implications of these strategies differ across countries. In regions with higher healthcare costs or limited resources for colonoscopy and pathologists, adopting such strategies that prioritize optical diagnosis may reduce the financial burden on the healthcare system. Nevertheless, each patient should still reserve the right to make informed choices on the treatment of their colon polyps. In regions where colonoscopy is more available and affordable (57), the “detect more, resect all and evaluate more” might be a more suitable strategy.

We acknowledge several limitations in our study. Firstly, being a single-center study with a patient population limited to Asians, the generalizability of our findings may be constrained by the relatively confined genetic and population background. Secondly, a more comprehensive understanding could be achieved through subgroup analysis of different segments of the colon, thereby enhancing clinical relevance. Lastly, future research endeavors will explore potential treatment management strategies tailored to different sizes and/or segments of polyps during their progression toward malignancy.

In summary, this study seeks to reassess the overall CRC risk by considering the entire polyp population, offering a comprehensive perspective and anticipating that our research may contribute to a fresh understanding of colorectal cancer screening and prevention strategies. The results of the study underscore the significance of small and diminutive polyps in the context of CRC development and the potential of considering the total number of small and diminutive polyps as an indicator of HGD incidences. In clinical practice, endoscopists need to weigh the combined risk of HGD from all small and diminutive polyps, ensuring a holistic evaluation to minimize the risk of CRC progression. Future research can focus on region-specific cost–benefit analyses as well as the relationship between the progression of cancer and the size of HGD.

## Data availability statement

The original contributions presented in the study are included in the article/**Supplementary Material**, further inquiries can be directed to the corresponding author.

## Author contributions

JZ: Conceptualization, Methodology, Writing – original draft, Writing – review & editing, Supervision. HS: Conceptualization, Methodology, Supervision, Writing – original draft, Writing – review & editing, Data curation. FX: Conceptualization, Methodology, Writing – original draft, Writing – review & editing. SL: Conceptualization, Methodology, Writing – review & editing. GZ: Methodology, Writing – review & editing, Project administration. XX: Methodology, Writing – review & editing. LL: Writing – review & editing. PW: Writing – review & editing, Conceptualization, Data curation, Methodology, Writing – original draft.

## Supplementary material

Supplementary Figure 1 The relationship between the size, morphology, location, histological subtype of polyps, and the quantity of HGD.

Supplementary Figure 2 Epidemic risk factors for HGD polyps. Age over 45 and a family history of colorectal cancer are associated with HGD number. *HGD, High-grade dysplasia.
