# Supplementary material for: The absolute number of small and diminutive adenomas with high-grade dysplasia is substantially higher compared with large adenomas: a retrospective pooled study
Source: Front Oncol. 2024 Feb 12;14:1294745. doi: 10.3389/fonc.2024.1294745 (PMC10896556; doi:10.3389/fonc.2024.1294745)
Supplement: Supplementary file 1 [file Table_1.docx]

**Supplementary tables**

**Table S1**. **Paris classification of polyps**

| Characteristics | **Entire Colon** | | | **Rectosigmoid Colon** | | |
| --- | --- | --- | --- | --- | --- | --- |
|  | Diminutive Polyp  (1-5 mm), % | Small Polyp  (6-9 mm), % | Large Polyp  (≥10mm), % | Diminutive Polyp  (1-5 mm), % | Small Polyp  (6-9 mm), % | Large Polyp  (≥10mm), % |
| Ⅰs | 1.65 (35/2118) | 2.22 (8/361) | 19.57 (9/46) | 1.57 (15/957) | 3.15 (4/127) | 28.00(7/25) |
| Ⅰsp | 19.05 (4/21) | 10.59 (9/85) | 37.14 (13/35) | 21.43 (3/14 | 9.09 (4/44) | 44.00(11/25) |
| Ⅰp | 0.00 (0/4) | 4.17(1/24) | 11.11 (4/36) | 0.00 (0/1) | 0.00(0/8) | 8.33 (2/24） |

**Table S2. Composition ratio and individual risk of small/diminutive HGD polyps**

| Characteristics | **Entire Colon** | | | **Rectosigmoid Colon** | | |
| --- | --- | --- | --- | --- | --- | --- |
|  | Diminutive Polyp  (1-5 mm), % | Small Polyp  (6-9 mm), % | P value* | Diminutive Polyp  (1-5 mm), % | Small Polyp  (6-9 mm), % | P value* |
| Composition ratio | 68.42 (39/57) | 31.58 (18/57) | 0.0002 | 69.23 (18/26) | 30.27 (8/26) | 0.0126 |
| Individual Risk of HGD | 1.82(39/2143) | 3.83 (18/470) | 0.0115 | 5.08 (18/354) | 5.63 (8/142) | 0.9799 |

*P value from two sample proportion tests.
