# Supplementary figures and images for: The absolute number of small and diminutive adenomas with high-grade dysplasia is substantially higher compared with large adenomas: a retrospective pooled study
Source: Front Oncol. 2024 Feb 12;14:1294745. doi: 10.3389/fonc.2024.1294745 (PMC10896556; doi:10.3389/fonc.2024.1294745)

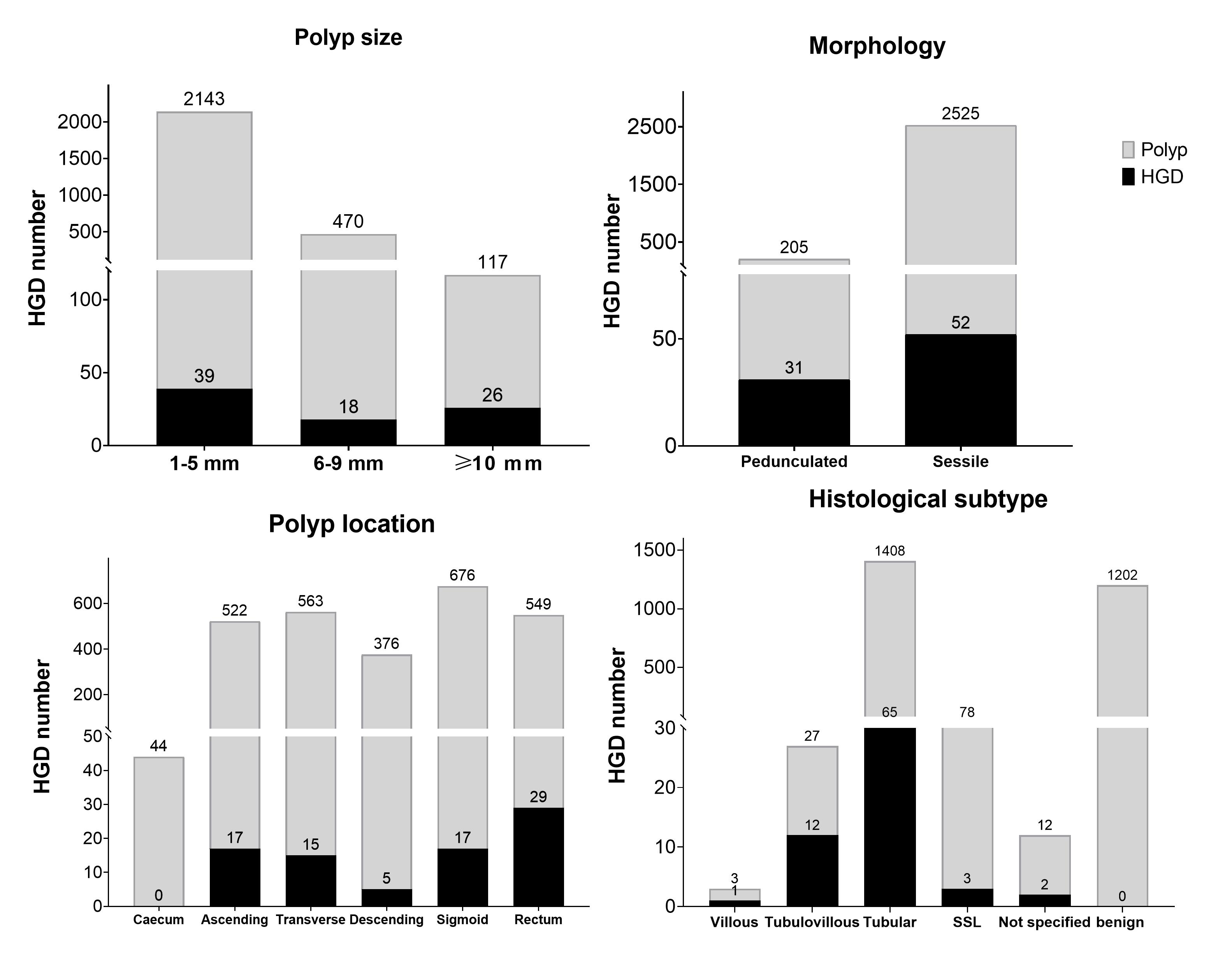

Supplement: Supplementary Figure 1 — The relationship between the size, morphology, location, histological subtype of polyps, and the quantity of HGD. [file Image_1.jpeg]

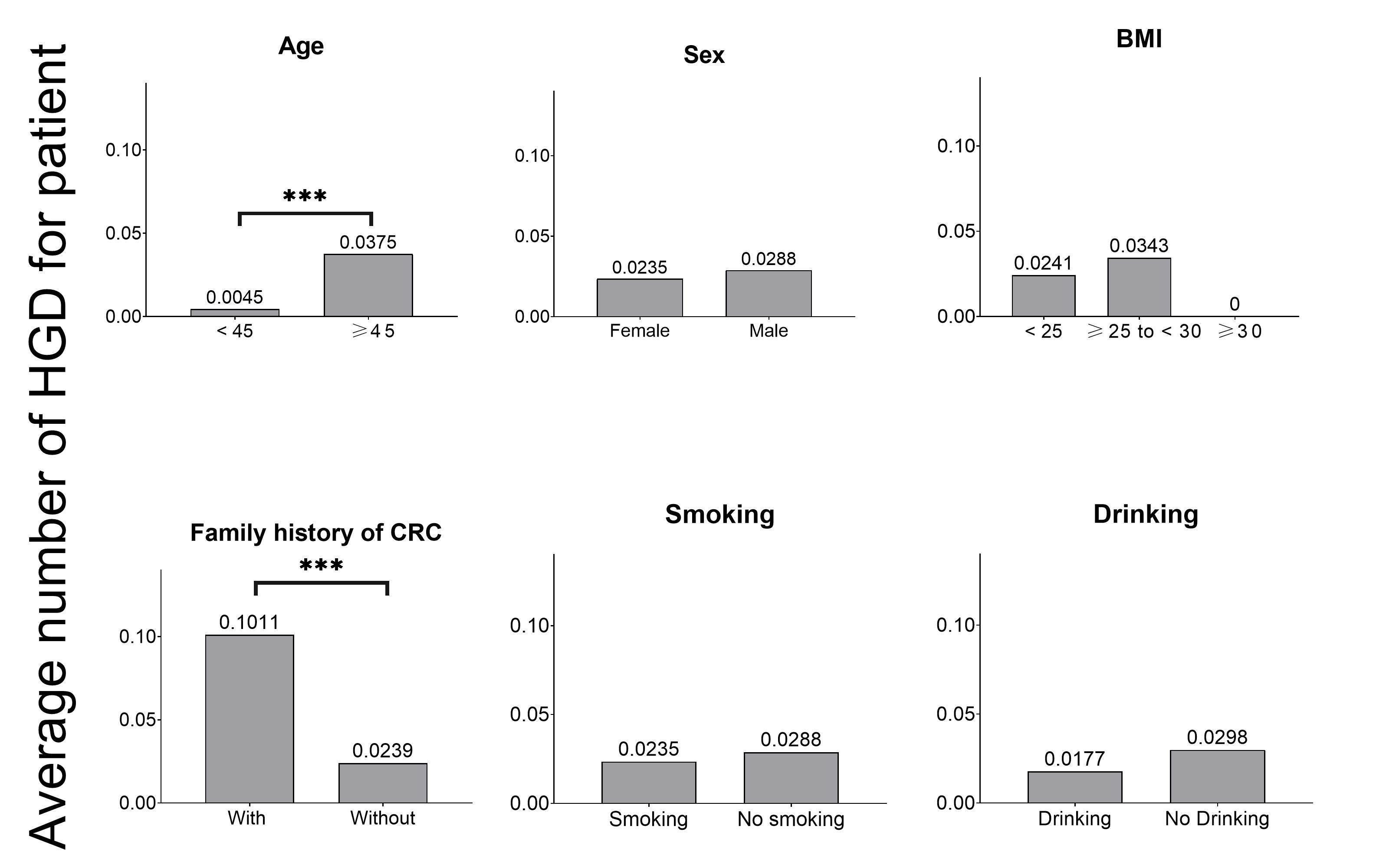

Supplement: Supplementary Figure 2 — Epidemic risk factors for HGD polyps. Age over 45 and a family history of colorectal cancer are associated with HGD number. *HGD, High-grade dysplasia. [file Image_2.jpeg]
